# Management of serum phosphorus over a 1-year follow-up in patients on peritoneal dialysis prescribed sucroferric oxyhydroxide as part of routine care: a retrospective analysis

**DOI:** 10.1186/s12882-024-03633-8

**Published:** 2024-06-17

**Authors:** Kamyar Kalantar-Zadeh, Linda H. Ficociello, Meijiao Zhou, Michael S. Anger

**Affiliations:** 1 Division of Nephrology and Hypertension and Transplantation, Harbor-UCLA Medical Center and The Lundquist Institute, Torrance, CA USA; 2 Fresenius Medical Care, Global Medical Office, Waltham, MA USA

**Keywords:** Sucroferric oxyhydroxide, Peritoneal dialysis, Phosphate binder, Pill burden, Hyperphosphatemia, Phosphorus

## Abstract

**Background:**

Hyperphosphatemia is associated with increased morbidity and mortality in patients with end-stage kidney disease (ESKD). Whereas clinical and observational studies have demonstrated the effectiveness of sucroferric oxyhydroxide (SO) in controlling serum phosphorus (sP) in ESKD, data on the real-world impact of switching to SO in patients on peritoneal dialysis (PD) are limited. In this retrospective database analysis, we examine the impact of SO on sP management over a 1-year period among PD patients prescribed SO as part of routine clinical care.

**Methods:**

We analyzed de-identified data from adults on PD in Fresenius Kidney Care clinics who were prescribed SO monotherapy between May 2018 and December 2019 as part of routine clinical management. Changes from baseline in sP levels, phosphate binder (PB) pill burden, and laboratory parameters were evaluated during the four consecutive 91-day intervals of SO treatment.

**Results:**

The mean age of the 402 patients who completed 1 year of SO was 55.2 years at baseline, and they had been on PD for an average of 19.9 months. SO was initiated with no baseline PB recorded in 36.1% of patients, whereas the remaining 257 patients were switched to SO from sevelamer (39.7%), calcium acetate (30.4%), lanthanum (1.2%), ferric citrate (14.0%), or more than one PB (14.8%). Mean sP at baseline was 6.26 mg/dL. After being prescribed SO, the percentage of patients achieving sP ≤ 5.5 mg/dL increased from 32.1% (baseline) to 46.5–54.0% during the 1-year follow-up, whereas the mean number of PB pills taken per day decreased from 7.7 at baseline (among patients on a baseline PB) to 4.6 to 5.4. Serum phosphorus and PB pill burden decreased regardless of changes in residual kidney function over the 12-month period. Similar results were observed for the full cohort (976 patients who either completed or discontinued SO during the 1-year follow-up).

**Conclusions:**

Patients on PD who were prescribed SO as part of routine care for phosphorus management experienced significant reductions in SP and PB pills per day and improvements in sP target achievement, suggesting the effectiveness of SO on SP management with a concurrent reduction in pill burden.

**Supplementary Information:**

The online version contains supplementary material available at 10.1186/s12882-024-03633-8.

## Background

The use of peritoneal dialysis (PD) in patients with end-stage kidney disease (ESKD) is increasing in the United States and in many countries throughout the world [1, 2]. Phosphorus metabolism is frequently disrupted in patients with ESKD, leading to hyperphosphatemia, which can result in cardiovascular and bone complications and increased mortality [3].

Longitudinal data from the Peritoneal Dialysis Outcomes and Practice Patterns Study (PDOPPS), an international prospective cohort study of 5487 patients on PD, reported that 37% of patients had serum phosphorus (sP) > 5.5 mg/dL and 67% had sP > 4.5 mg/dL [4]. In this study, higher sP levels were a strong predictor of all-cause mortality and cardiovascular morbidity, with adjusted hazard ratios for all-cause mortality of 1.53 (95% confidence interval [CI], 1.14–2.05) and 1.19 (95% CI, 0.92–2.05) for patients with baseline sP of ≥ 6.5 mg/dL and 5.5 to < 6.5 mg/dL, respectively (relative to baseline sP of ≥ 3.5 to ≤ 4.5 mg/dL). To mitigate these risks, current Kidney Disease: Improving Global Outcomes (KDIGO) guidelines recommend targeting sP levels toward the normal range in patients with kidney disease through dietary modification, dialysis intensification, and/or the use of phosphate binders (PBs) [5].

Although patients receiving PD often have higher levels of residual kidney function [6] and generally have lower sP levels than patients receiving hemodialysis [7–9], treatment with oral PBs is frequently indicated [4]. PBs account for approximately half the pill burden in patients on maintenance dialysis, which frequently exceeds 20 pills per day [10]. As such, PBs that effectively reduce sP with a lower pill burden, minimal side effects, and a lower cost may be preferred by clinicians and patients alike.

Clinical and observational studies have shown the effectiveness of the iron-based, non-calcium, chewable PB, sucroferric oxyhydroxide (SO; VELPHORO®, Fresenius Medical Care Renal Therapies Group, Waltham, MA, USA) in controlling sP in patients with ESKD on PD [11–13]. A real-world retrospective analysis of patients with PD prescribed SO for 6 months demonstrated an association between SO initiation and lower sP, improved sP target achievement, and reduced PB pill burden [12]. In this retrospective analysis, we aimed to examine changes in sP and PB pill burden over a 1-year period in patients on PD initiating SO, after either no recorded use of PB or switching from another PB.

## Methods

### Data source and study population

To conduct this retrospective analysis, we extracted de-identified medical records and prescription data from the Fresenius Kidney Care (FKC) clinical data warehouse and the FreseniusRx pharmacy database. Adult FKC patients aged 18 years and older who dialyzed with PD and received SO monotherapy between May 2018 and December 2019 as part of their routine care were included in the analysis. To be eligible for inclusion, patients must have had sP results available from the 3 months before SO therapy was initiated (full cohort). Patients who received uninterrupted SO prescriptions (i.e., no gaps in SO prescriptions of more than 45 continuous days) for at least 1 year made up the completers cohort. Observation periods were defined in consecutive 91-day quarterly (Q) intervals, including a baseline period (3 months before the SO prescription; –Q1) and four consecutive intervals of SO treatment during the follow-up period (Q1–Q4). This study was reviewed by an independent review board (New England Independent Review Board (NEIRB)/WCG IRB, Needham, MA, USA), and was granted an exempt status determination under the common rule and applicable guidance because of its purely observational nature and use of only de-identified data.

### Data assessments

Patient-level demographic characteristics, including age, sex, race, ethnicity, dialysis vintage, primary cause of kidney failure, prevalence of comorbidities, PB use, and PD modality (i.e., continuous ambulatory PD [CAPD], automated PD [APD], or a mix between both) were evaluated at baseline. Clinical and laboratory parameters evaluated included serum mineral and bone disorder (MBD) markers (phosphorus, calcium, intact parathyroid hormone [iPTH]), nutritional and dialytic clearance parameters (serum albumin, phosphorus-attuned albumin, normalized protein catabolic rate [nPCR], PD Kt/V, and dialysis Kt/V). Phosphorus-attuned albumin was calculated by dividing serum albumin by sP [12]. Corrected serum calcium was calculated using Orrell’s formula to account for albumin binding [14]. Laboratory tests were conducted monthly, except for iPTH, which was measured quarterly per standard practice at FKC facilities, and analyzed at a central laboratory (Spectra Laboratories, Rockleigh, NJ, USA).

Renal urea clearance (as a measure of residual kidney function [K_ru_]) was assessed, and changes in categorical K_ru_ (no K_ru_; K_ru_ ≤3 mL/min; K_ru_ >3 mL/min) across the study period were categorized into three groups: those with no K_ru_ during the entire study period, those whose K_ru_ decreased from baseline to Q4, and those whose K_ru_ did not change from baseline to Q4. Reductions in K_ru_ from baseline to Q4 were further calculated by the values of K_ru_ and categorized by the magnitude of decrease (≤ 1 mL/min, 1–3 mL/min, and > 3 mL/min). The use of ESKD-related medications, including PB binders, cinacalcet, and vitamin D, were evaluated quarterly, as was the proportion of patients below the upper sP limit of 5.5 mg/dL (per National Kidney Foundation Kidney Disease Outcomes Quality Initiative [NKF KDOQI] recommendations) and below 4.5 mg/dL (based on KDIGO guidelines).

### Statistical analysis

Baseline characteristics are presented as the mean (standard deviation [SD]) for continuous variables and as the number of patients with percentages for categorical variables. Quarterly arithmetic means for continuous data were calculated, and the overall significance tests were conducted using mixed-effects linear regression. Cochran’s Q test was used to evaluate the statistical significance of differences in categorical variables over time. Two-tailed *P* values < 0.05 were considered statistically significant. Most analyses were conducted for both the completers cohort and full cohort, with data after patients discontinued SO or switched to hemodialysis being censored in the full cohort. Subgroup analyses defined by K_ru_ changes were performed only for the completers cohort; this was because of challenges in defining K_ru_ changes among patients who discontinued SO, as a result of significant loss to follow-up toward the end of the study. Analyses performed for the completers cohort are considered the main analyses and are the primary focus of the results sections. An additional analysis was conducted in another group of PD patients who were prescribed non-SO monotherapy (sevelamer, calcium acetate, lanthanum carbonate, or ferric citrate) between May 2018 and December 2019 and followed up for one year with the same binder.

All analyses were conducted with SAS (SAS Enterprise Guide 8.3; SAS Institute Inc., Cary, NC, USA).

## Results

### Demographics and patient characteristics

**Fig. 1:**
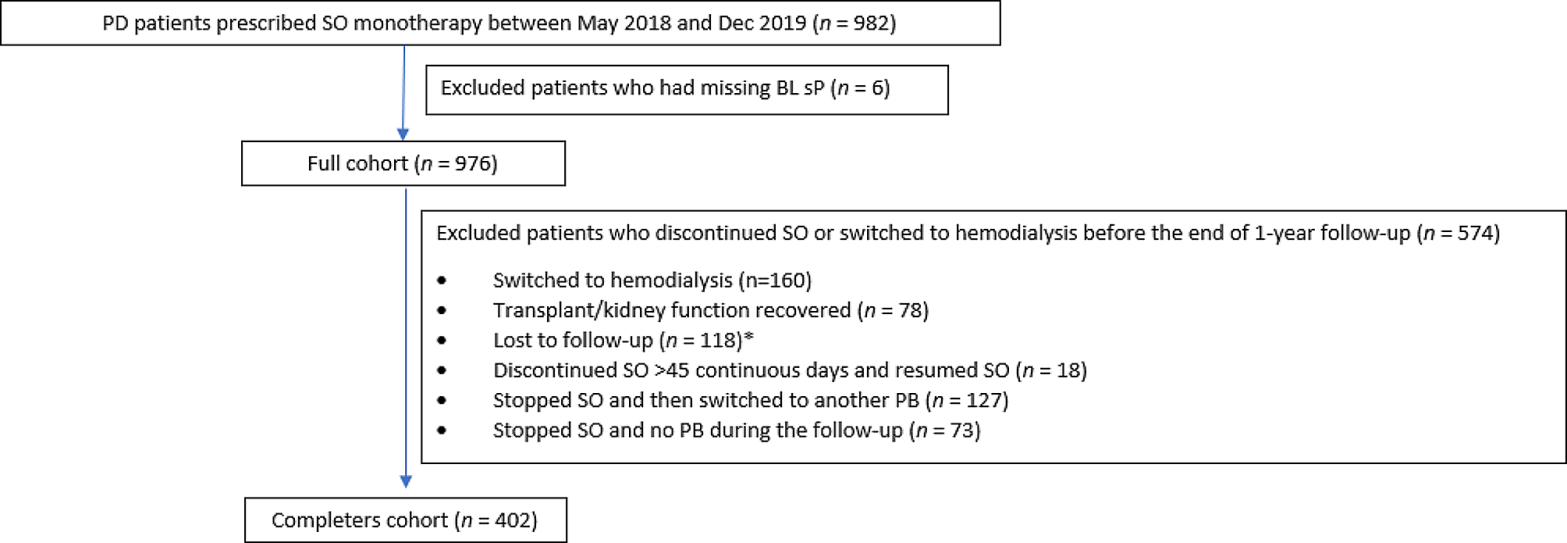
Patient disposition. *Included patients who left the country (*n* = 5), transferred to a non-FMC facility (*n* = 31), or died (*n* = 82). PD, peritoneal dialysis; SO, sucroferric oxyhydroxide; BL, baseline; sP, serum phosphorus; PB, phosphate binder; FMC = Fresenius Medical Care

Of the 982 patients prescribed SO monotherapy, 6 patients had a missing sP assessment at baseline, leaving 976 patients in the full cohort. A total of 574 patients discontinued SO or switched to hemodialysis before the end of the 1-year follow-up, leaving 402 patients in the main analysis set (completers cohort; Fig. 1).

Demographic and baseline characteristics of the completers cohort are shown in Table 1. The mean (SD) age was 55.2 (12.9) years and the mean (SD) dialysis vintage was 19.9 (29.5) months. Women accounted for 43.3% of the analysis population. Most patients (60.4%) were white and 13.4% were Hispanic. Diabetes was the most common cause of kidney failure, accounting for 40% of cases. More than one-third of patients (36.1%) did not have recorded use of a PB at baseline, although the use of over-the-counter PBs cannot be excluded. Among patients who had recorded use of a PB at baseline, sevelamer was the most prescribed (39.7%), followed by calcium acetate (30.4%) and ferric citrate (14.0%). More than three-quarters of patients (77.9%) were receiving APD at baseline.

**Table 1:** Baseline characteristics of the study population: completers cohort and full cohort

Characteristic	Completers Cohort (*N* = 402)	Full Cohort (*N* = 976)
Age, yr, mean ± SD	55.2 ± 12.9	54.8 ± 13.6
Dialysis vintage, months, mean ± SD	19.9 ± 29.5	20.2 ± 26.5
Female, *n* (%)	174 (43.3)	434 (44.5)
Race, *n* (%)		
White	243 (60.4)	575 (58.9)
Black/African American	112 (27.9)	233 (23.9)
Other	17 (4.2)	42 (4.3)
Unknown	30 (7.5)	126 (12.9)
Hispanic/Latino, *n* (%)	54 (13.4)	107 (11.0)
Primary cause of kidney failure, *n* (%)		
Diabetes mellitus	161 (40.0)	417 (42.7)
Hypertension	141 (35.1)	284 (29.1)
Glomerulonephritis	46 (11.4)	118 (12.1)
Polycystic kidney	14 (3.5)	43 (4.4)
Other	40 (10.0)	114 (11.7)
Comorbidities, *n* (%)		
Diabetes mellitus	183 (45.5)	471 (48.3)
Congestive heart failure	19 (4.7)	73 (7.5)
Phosphate binder at baseline, *n* (%)		
No PB recorded	145 (36.1)	313 (32.1)
PB recorded	257 (63.9)	663 (67.9)
Sevelamer	102 (39.7)	285 (43.0)
Calcium acetate	78 (30.4)	178 (26.8)
Lanthanum carbonate	3 (1.2)	15 (2.3)
Ferric citrate	36 (14.0)	84 (12.7)
> 1 PB recorded	38 (14.8)^a^	101 (15.2)^c^
PD modality, *n* (%)		
CAPD	48 (11.9)	113 (11.6)
APD	313 (77.9)	778 (79.7)
Switched between CAPD and APD	41 (10.2)^b^	85 (8.7)^d^

^a^Among these patients, 23 (61%) patients received monotherapy; ^b^Among 41 patients, 37 (90%) patients switched from CAPD to APD and 4 (10%) switched from APD to CAPD; ^c^Among these patients, 61 (60%) patients received monotherapy; ^d^Among 85 patients, 76 (89%) patients switched from CAPD to APD and 9 (11%) switched from APD to CAPD. SD, standard deviation; PB, phosphate binder; PD, peritoneal dialysis; CAPD, continuous ambulatory peritoneal dialysis; APD, automated peritoneal dialysis

The baseline characteristics of the full cohort (Table 1) were generally similar to those reported for the completers cohort. Relative to the completers cohort, slightly higher rates of comorbidities (diabetes: 48.3% vs. 45.5%; heart failure: 7.5% vs. 4.7%) and prior PB use at baseline (67.9% vs. 63.9%) were observed in the full cohort.

### Changes in serum phosphorus and pill burden

**Fig. 2:**
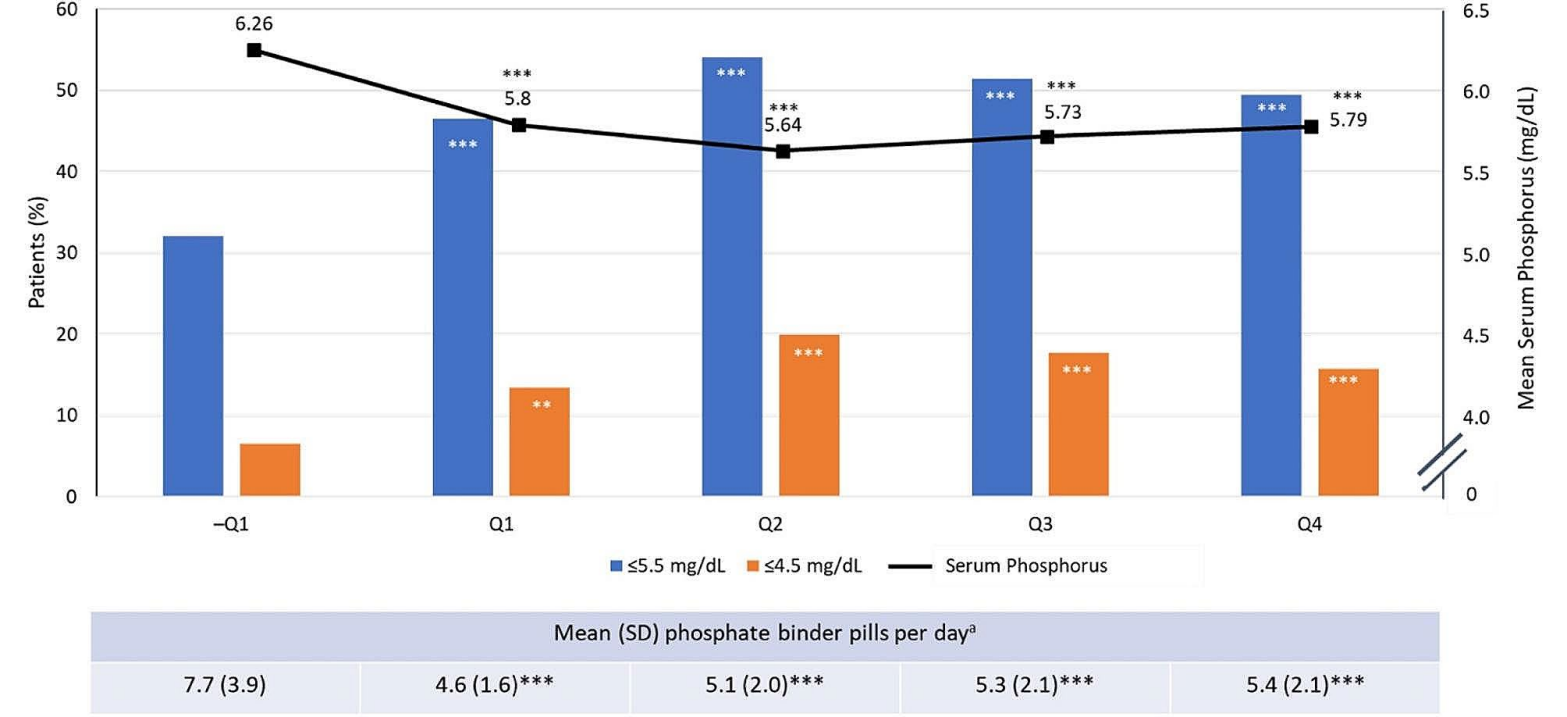
sP levels, proportion of patients achieving in-range sP, and daily PB pill burden during study period among completers cohort. *P < 0.05; **P < 0.001; ***P < 0.0001 (vs. baseline). ^a^Pill burden calculated for patients with non–sucroferric oxyhydroxide PB recorded at baseline. Q, quarter; SD, standard deviation; sP, serum phosphorus; PB, phosphate binder

At baseline, the mean (SD) sP among patients in the completers cohort was 6.3 (1.4), and 32.1% of patients had sP ≤ 5.5 mg/dL prior to initiating SO. Fewer than 7% of patients had baseline sP levels of ≤ 4.5 mg/dL. After starting SO, mean sP significantly decreased throughout the 1-year follow-up period (*P* < 0.0001), reaching a nadir of 5.6 (1.4) in Q2 (*P* < 0.0001; Fig. 2). The percentage of patients achieving sP ≤ 5.5 mg/dL was 46.5% in Q1, 54.0% in Q2, 51.4% in Q3, and 49.4% in Q4 (*P* < 0.0001 vs. baseline for all comparisons). The proportions of patients achieving the more stringent target of sP ≤ 4.5 mg/dL were 13.4%, 19.9%, 17.7%, and 15.7%, respectively (*P* < 0.001 for Q1 and *P* < 0.0001 for Q2–Q4 vs. baseline; Fig. 2).

At baseline, among those patients receiving a PB prior to SO, the mean (SD) daily PB pill burden was 7.7 (3.9). Daily PB pill burden was significantly lower while patients were on SO, ranging from 4.6 (1.6) pills/day in Q1 to 5.4 (2.1) pills/day in Q4 (*P* < 0.0001; Fig. 2).

### Changes in serum phosphorus and pill burden by residual kidney function

Those patients in the completers cohort with no residual kidney function throughout the study period had the highest baseline sP (6.8 mg/dL) despite a PB pill burden (among those receiving a PB) of more than 10 pills per day. Overall, K_ru_ decreased from 3.99 mL/min to 3.37 mL/min over the 1-year follow-up period (*P* < 0.0001; Table 2). Among patients in the completers cohort, 6.7% had a K_ru_ of 0 mL/min throughout the study, whereas 64.4% exhibited no change in K_ru_ category and 28.9% demonstrated reductions in K_ru_ category. Among those with reductions in K_ru_, the magnitude of reduction was most commonly 1 to 3 mL/min during the follow-up period. Significant reductions in sP and PB pill burden were observed at each follow-up period across each of the main K_ru_ subgroups (Table 3).

**Table 2:** Comparison of quarterly changes in clinical parameters and CKD-MBD medication use among completers cohort

Parameters	Baseline	Follow-up				*P*-value
	–1Q; ref (*n* = 402)	Q1 (*n* = 402)	Q2 (*n* = 402)	Q3 (*n* = 401)	Q4 (*n* = 401)	
CKD-MBD biochemical markers						
sP, mg/dL	6.26 ± 1.39	5.80 ± 1.36	5.64 ± 1.37	5.73 ± 1.38	5.79 ± 1.41	< 0.0001
sP ≤ 5.5 mg/dL, %	32.1	46.5	54.0	51.4	49.4	< 0.0001
sP ≤ 4.5 mg/dL, %	6.5	13.4	19.9	17.7	15.7	< 0.0001
Calcium, mg/dL	8.99 ± 0.68	8.92 ± 0.65	8.91 ± 0.63	8.87 ± 0.67	8.86 ± 0.68	< 0.0001
Corrected calcium, mg/dL	9.52 ± 0.68	9.46 ± 0.64	9.44 ± 0.63	9.41 ± 0.66	9.39 ± 0.68	< 0.0001
iPTH, pg/mL	448 ± 359	477 ± 372	480 ± 369	480 ± 338	478 ± 353	0.14
CKD-MBD medications						
PB pills/day^a^	7.7 ± 3.9	4.6 ± 1.6	5.1 ± 2.0	5.3 ± 2.1	5.4 ± 2.1	< 0.0001
Cinacalcet use, %	18.2	22.9	26.4	28.9	30.6	< 0.0001
Oral active vitamin D use, %	58.0	61.7	65.7	65.4	62.2	0.0002
Nutritional and clearance parameters						
Serum albumin, g/dL	3.67 ± 0.40	3.63 ± 0.39	3.62 ± 0.38	3.59 ± 0.40	3.59 ± 0.40	< 0.0001
Phosphorus-attuned albumin, × 10³	0.62 ± 0.16	0.67 ± 0.17	0.69 ± 0.172	0.67 ± 0.17	0.67 ± 0.17	< 0.0001
nPCR, g/kg/day	1.07 ± 0.28	1.07 ± 0.28	1.05 ± 0.29	1.05 ± 0.32	1.04 ± 0.28	0.13
PD Kt/V, dialysis + K_ru_	2.26 ± 0.90	2.26 ± 0.62	2.21 ± 0.59	2.19 ± 0.62	2.19 ± 0.55	0.17
Dialysis Kt/V	1.39 ± 0.42	1.42 ± 0.50	1.42 ± 0.38	1.47 ± 0.57	1.48 ± 0.49	< 0.0001
K_ru_, mL/min	3.99 ± 5.53	3.79 ± 2.57	3.75 ± 2.79	3.41 ± 2.82	3.37 ± 2.74	< 0.0001

Values are presented as arithmetic mean ± SD for continuous variables. Sample sizes are for patients with available sP measurements

CKD-MBD, chronic kidney disease–mineral and bone disorder; Q, quarter; sP, serum phosphorus; iPTH, intact parathyroid hormone; PB, phosphate binder; nPCR, normalized protein catabolic rate; PD, peritoneal dialysis; K_ru_, residual kidney function

**Table 3:** sP levels and pill burden in subgroups defined by K_ru_ changes from baseline to 12 months (Q4) and magnitude of those change (completers cohort)

Parameter	Baseline	Follow-up				*P*-value
	–1Q; ref	Q1	Q2	Q3	Q4	
**No K**_**ru**_ **during study period (n = 27)**						
PB pills/day^a^	10.2 ± 4.8	4.7 ± 1.5	5.0 ± 1.5	5.2 ± 1.6	5.3 ± 1.8	< 0.0001
sP, mg/dL	6.83 ± 1.63	6.44 ± 1.44	5.95 ± 1.77	6.08 ± 1.60	5.88 ± 1.49	< 0.0001
sP ≤ 5.5 mg/dL, %	22.2	22.2	48.1	40.7	48.1	0.006
sP ≤ 4.5 mg/dL, %	7.4	7.4	25.9	14.8	11.1	0.04
**K**_**ru**_**no change from BL to Q4 (*n*** **= 259)**						
PB pills/day^a^	7.1 ± 3.5	4.4 ± 1.7	4.9 ± 2.0	5.1 ± 2.1	5.2 ± 2.2	< 0.0001
sP, mg/dL	6.08 ± 1.27	5.62 ± 1.30	5.43 ± 1.26	5.53 ± 1.19	5.57 ± 1.19	< 0.0001
sP ≤ 5.5 mg/dL, %	35.5	51.4	60.2	57.1	53.3	< 0.0001
sP ≤ 4.5 mg/dL, %	6.9	16.2	23.9	18.9	18.1	< 0.0001
**K**_**ru**_**decreased from BL to Q4 (*n*** **= 116)**						
PB pills/day^a^	8.2 ± 4.0	4.9 ± 1.7	5.5 ± 2.1	5.7 ± 2.0	5.8 ± 2.0	< 0.0001
sP, mg/dL	6.51 ± 1.53	6.05 ± 1.40	6.05 ± 1.41	6.10 ± 1.64	6.26 ± 1.71	< 0.0001
sP ≤ 5.5 mg/dL, %	26.7	41.4	41.4	40.9	40.9	0.01
sP ≤ 4.5 mg/dL, %	5.2	8.6	9.5	15.7	11.3	0.06
*K*_*ru*_ *decreased by ≤ 1 mL/min from BL to Q4 (n = 25)*						
PB pills/day^a^	9.3 ± 5.8	4.8 ± 1.6	5.5 ± 2.2	5.9 ± 2.3	5.9 ± 2.2	< 0.0001
sP, mg/dL	7.38 ± 1.65	6.31 ± 1.30	6.09 ± 1.53	5.99 ± 1.27	6.26 ± 1.63	< 0.0001
sP ≤ 5.5 mg/dL, %	12.0	32.0	48.0	33.3	41.7	0.005
sP ≤ 4.5 mg/dL, %	4.0	4.0	12.0	12.5	12.5	0.62
*K*_*ru*_ *decreased by 1–3 mL/min from BL to Q4 (n = 54)*						
PB pills/day^a^	7.6 ± 2.6	4.8 ± 1.7	5.5 ± 2.3	5.7 ± 2.1	5.7 ± 2.0	< 0.0001
sP, mg/dL	6.45 ± 1.48	6.02 ± 1.21	5.95 ± 1.15	5.95 ± 1.47	6.07 ± 1.52	0.009
sP ≤ 5.5 mg/dL, %	20.4	35.2	33.3	40.7	37.0	0.09
sP ≤ 4.5 mg/dL, %	5.6	7.4	9.3	16.7	14.8	0.17
*K*_*ru*_ *decreased by > 3 mL/min from BL to Q4 (n = 37)*						
PB pills/day^a^	7.8 ± 3.4	5.1 ± 1.7	5.2 ± 1.6	5.6 ± 1.6	5.8 ± 1.9	< 0.0001
sP, mg/dL	6.00 ± 1.28	5.92 ± 1.70	6.18 ± 1.69	6.38 ± 2.04	6.53 ± 2.00	0.0001
sP ≤ 5.5 mg/dL, %	45.9	56.8	48.6	45.9	45.9	0.69
sP ≤ 4.5 mg/dL, %	5.4	13.5	8.1	16.2	5.4	0.26

Values are presented as arithmetic mean ± SD for continuous variables

^a^Pill burden calculated for patients with non-SO PB recorded at BL. Categorical K_ru_ was used to define the trend from BL to Q4: 0, no K_ru_; 1, K_ru_ ≤3 mL/min; 2, K_ru_ >3 mL/min; values of K_ru_ were used to calculate the magnitude. sP, serum phosphorus; K_ru_, residual kidney function; Q, quarter; PB, phosphate binder; BL, baseline; SD, standard deviation; SO, sucroferric oxyhydroxide

### Other chronic kidney disease–mineral and bone disorder parameters

Over the 1-year follow-up period, statistically significant decreases from baseline in calcium (a mean decrease of up to 0.13 mg/dL in Q4; *P* < 0.0001 overall) and corrected calcium (a mean decrease of 0.13 mg/dL in Q4; *P* < 0.0001) were observed in the completers cohort. Numerical increases in mean iPTH level were observed but these changes were not statistically significant overall. The percentage of patients with reported cinacalcet use progressively increased from 18.2% at baseline to 30.6% at Q4 (*P* < 0.0001). Small but significant increases in the proportion of patients using oral active vitamin D were observed over the follow-up period (*P* = 0.0002). A detailed presentation of these parameters is included within Table 2.

### Nutritional and clearance parameters

Following the initiation of SO, mean serum albumin levels decreased from 3.67 (baseline) to 3.59 g/dL at Q3 and Q4 (*P* < 0.0001 overall) in the completers cohort. After adjustment of albumin levels for phosphorus concentrations (i.e., phosphorus-attuned albumin level), SO initiation was associated with mean increases of 0.05 × 10^3^ to 0.07 × 10^3^ (*P* < 0.0001 overall). Dietary protein intake, as assessed by nPCR, and overall PD Kt/V remained consistent throughout the follow-up period. Dialysis adequacy increased progressively during follow-up, whereas residual kidney function (assessed by K_ru_) progressively deteriorated (*P* < 0.0001 for both; Table 2).

### Full cohort analysis

Relative to the completers cohort, the full cohort had a higher mean baseline sP (6.45 vs. 6.26 mg/dL), lower mean baseline serum calcium (8.90 vs. 8.99 mg/dL), higher mean baseline iPTH (480 vs. 448 pg/mL), and lower K_ru_ (3.56 vs. 3.99 mL/min). Nonetheless, the inclusion of patients who discontinued SO or switched to hemodialysis during the 12-month follow-up period did not appear to impact the effects associated with SO treatment. Significant reductions in sP and significant increases in the proportion of patients achieving prespecified sP cutoffs were observed during follow-up (Table 4). As observed in the completers cohort, SO treatment was also associated with significant reductions in daily PB pill burden, significant increases in phosphorus-attuned albumin, and significant increases in adequacy of dialysis as assessed by dialysis Kt/V. Increases in cinacalcet use were also observed across the follow-up period.

**Table 4:** Comparison of quarterly changes in clinical parameters and CKD-MBD medication use among full cohort

Parameters	Baseline	Follow-up				*P*-value
	–1Q; ref (*n* = 976)	Q1 (*n* = 960)	Q2 (*n* = 740)	Q3 (*n* = 604)	Q4 (*n* = 485)	
CKD-MBD biochemical markers						
sP, mg/dL	6.45 ± 1.43	6.18 ± 1.61	5.95 ± 1.55	5.90 ± 1.46	5.89 ± 1.47	< 0.0001
sP ≤ 5.5 mg/dL, %	26.2	38.5	45.9	45.4	46.2	< 0.0001
sP ≤ 4.5 mg/dL, %	4.2	10.8	16.5	15.9	15.3	< 0.0001
Calcium, mg/dL	8.90 ± 0.70	8.85 ± 0.68	8.84 ± 0.68	8.84 ± 0.67	8.85 ± 0.68	< 0.0001
Corrected calcium, mg/dL	9.44 ± 0.69	9.39 ± 0.68	9.38 ± 0.67	9.38 ± 0.67	9.38 ± 0.68	< 0.0001
iPTH, pg/mL	480 ± 381	505 ± 371	494 ± 376	501 ± 384	493 ± 373	0.12
CKD-MBD medications						
PB pills/day^a^	7.6 ± 3.8	4.5 ± 1.7	5.0 ± 1.9	5.2 ± 2.0	5.3 ± 2.1	< 0.0001
Cinacalcet use, %	19.8	23.9	26.0	27.4	29.2	< 0.0001
Oral active vitamin D use, %	57.0	60.6	64.0	64.5	62.5	0.0001
Nutritional and clearance parameters						
Serum albumin, g/dL	3.60 ± 0.42	3.56 ± 0.42	3.55 ± 0.44	3.54 ± 0.44	3.56 ± 0.42	< 0.0001
Phosphorus-attuned albumin, × 10³	0.59 ± 0.15	0.63 ± 0.18	0.65 ± 0.18	0.65 ± 0.18	0.65 ± 0.17	< 0.0001
nPCR, g/kg/day	1.04 ± 0.26	1.05 ± 0.27	1.04 ± 0.30	1.03 ± 0.31	1.03 ± 0.27	0.04
PD Kt/V, dialysis + K_ru_	2.20 ± 0.78	2.20 ± 0.58	2.19 ± 0.60	2.18 ± 0.62	2.17 ± 0.53	0.35
Dialysis Kt/V	1.42 ± 0.57	1.43 ± 0.47	1.43 ± 0.43	1.48 ± 0.56	1.48 ± 0.48	< 0.0001
K_ru_, mL/min	3.56 ± 4.05	3.50 ± 2.61	3.56 ± 2.74	3.37 ± 2.79	3.31 ± 2.71	< 0.0001

Values are presented as arithmetic mean ± SD for continuous variables. Sample sizes are for patients with available sP measurements. ^a^Pill burden calculated for patients with non-SO PB recorded at baseline. CKD-MBD, chronic kidney disease–mineral and bone disorder; Q, quarter; sP, serum phosphorus; iPTH, intact parathyroid hormone; PB, phosphate binder; nPCR, normalized protein catabolic rate; PD, peritoneal dialysis; K_ru_, residual kidney function; SO, sucroferric oxyhydroxide; SD, standard deviation

### Non-SO cohort analysis

Across FKC, a total of 2561 PD patients had a baseline sP greater than 5.5 mg/dL and were initiated on non-SO monotherapy between May 2018 and December 2019 and continued monotherapy with the same PB for at least one year. As detailed in supplemental Table 1, both SO and non-SO initiation were associated with reductions in sP, but patients prescribed SO monotherapy exhibited greater absolute reductions in sP.

## Discussion

In this real-world retrospective cohort study, PD patients prescribed SO as part of routine clinical care over a 1-year period experienced significant reductions in sP and PB pills per day, and increases in the percentage of patients who achieved sP ≤ 5.5 mg/dL or sP ≤ 4.5 mg/dL, suggesting improved sP management with a concurrent reduction in pill burden. These findings are consistent with our observations in a smaller cohort of patients on PD in 2014–2015 and the results of previous studies in patients on PD or hemodialysis [11–13, 15, 16]. Compared to our previous real-world analysis of SO initiation in patients on PD in 2014–2015, patients in the completers cohort of the current study were older (55.2 vs. 50.6 years) but were newer to dialysis (19.9 vs. 29.3 months) and had lower sP at baseline (6.26 vs. 6.59 mg/dL). In the prior study, the proportion of patients achieving ≤ 5.5 mg/dL increased by 72% from baseline to 6 months (vs. 68% in the completers cohort of the present analysis at Q2) and the prescribed number of PB pills per day decreased by 57% (vs. 34% in the present analysis at Q2) [12]. These benefits were sustained through the end of the 1-year follow-up period in the present study. Of note, in the present study, patients received slightly higher mean doses of SO at 6 months (5.1 vs. 4.9 pills/day) and doses were further titrated at 1 year (5.4 pills/day). Similar data were observed in the full cohort that included patients who discontinued SO prior to the end of the 1-year follow-up period.

Non-adherence to PBs is a common problem among patients on dialysis, reported in 36–62% of patients on dialysis [10, 17]. High pill burden is a well-known barrier to adherence in patients on dialysis, and evidence suggests that PB pill burden is inversely correlated with sP control [18–20]. A recent study in patients on hemodialysis found that inconvenience, difficulty, and dissatisfaction with PB medication were associated with poorer sP control (≥ 6 mg/dL) and a higher mortality risk [18], emphasizing the need to consider patient preference and satisfaction when selecting PB regimens.

Although the present study does not provide outcomes data, prior evidence has demonstrated that longer durations of sP control were independently associated with reduced mortality—a 3.2% decrease in risk for each additional month of sP control and a 38.1% decrease in risk for each additional year of sP control [21]. In that same study, a longer duration of sP control was also independently associated with lower rates of PD withdrawal. Together, these data reinforce the importance of PB regimens and the need to consider factors that may affect adherence and persistence in patients on PD.

Residual kidney function is a key factor impacting phosphate clearance and balance in patients on PD [6, 22] and can influence the perceived efficacy of PB. Reductions in K_ru_ have been significantly correlated with increases in sP [23]. In the present analysis, overall, K_ru_ among patients continuing SO for 1 year decreased from 3.99 mL/min at baseline to 3.37 mL/min at 1 year. A corresponding increase in dialysis Kt/V was also documented, suggesting clinicians adjusted PD prescriptions to compensate for decreasing kidney function. Patients whose K_ru_ did not change during the treatment period attained the greatest levels of sP control with the lowest sP (5.57 mg/dL) and the highest percentage of patients reaching sP targets (≤ 5.5 mg/dL: 53.3%; ≤4.5 mg/dL: 18.1% ). These patients were also prescribed the fewest PB pills per day at Q4 (5.2 pills/day). In contrast, patients whose K_ru_ decreased by > 3 mL/min had the highest sP at Q4 (6.53 mg/dL), compared to subgroups with smaller K_ru_ decreases. These data suggest the need for increased attention to PB dose titration in patients with the most rapid decreases in residual kidney function. They also support the potential importance of efforts to maintain residual kidney function and individualizing PD prescriptions to optimize phosphate management [22].

Typically, the management of phosphate levels in ESKD requires balancing the need to maintain or increase dietary protein intake while trying to avoid high levels of hyperphosphatemia [24]. In this study, increases in levels of phosphorus-attuned albumin in the context of no changes in nPCR suggest that SO reduced sP without adverse changes in nutritional status. It has been suggested that dietary intake may improve with SO vs. other binders, given the impact of regimens with higher pill burdens on appetite [16]. A recent pilot clinical practice study documented similar increases in phosphorus-attuned albumin but did not find any significant changes in patient-reported appetite or dietary intake among PD patients initiating SO [13].

This real-world evaluation of patients who switched to SO also found changes in multiple markers of MBD and MBD-related medications over the 1-year observation period. Serum levels of calcium and corrected calcium declined, a finding that may be explained, at least in part, by the discontinuation of calcium acetate in approximately 20% of patients at baseline. A non-significant increase in serum iPTH was also noted, despite an increase in the use of cinacalcet and oral active vitamin D. Together, these MBD-related findings may reflect the progression of hyperparathyroidism rather than the direct effects of SO.

Although this analysis provides important insight into the impact of SO therapy on hyperphosphatemia, pill burden, and MBD parameters in PD patients, it does have some limitations, including its retrospective study design and lack of a comparator group. The absence of information regarding the clinical rationale for switching to SO and/or reasons for discontinuation of other PBs hinders our ability to fully characterize the patient population receiving SO in clinical practice. Information on the safety and tolerability of SO and patient adherence with prescribed therapy are also lacking. Database analyses are also limited by the difficulty in determining whether changes in certain outcomes are related to the use of SO or sP control or could be attributed to increasing dialysis vintage and worsening underlying condition. It is also not possible to exclude the possibility that nutritional changes and/or alterations in other medications, including the observed increase in cinacalcet usage, contributed to observed changes in sP. Finally, although we present preliminary data examining sP reductions observed among patients initiated on non-SO PBs, it is inappropriate to draw comparative conclusions regarding the efficacy of different PBs.

## Conclusions

Results from this retrospective observational study demonstrate that patients on PD prescribed SO as part of routine care over a 1-year period experienced significant reductions in sP and PB pill burden and increased attainment of target sP levels, suggesting that SO improved sP management while reducing medication burden in PD. In combination with prior studies, these data suggest that initiating SO in PD patients with hyperphosphatemia may allow for increased phosphorus control with reduced pill burden and maintained or improved nutritional status.

### Electronic supplementary material

Supplementary material

## Data Availability

The data underlying the findings described in this manuscript are proprietary and not publicly available. Further inquiries can be directed to Dr. Michael S. Anger at Michael.Anger@freseniusmedicalcare.com.

## Abbreviations

APD: Automated peritoneal dialysis
CAPD: Continuous ambulatory peritoneal dialysis
CI: Confidence interval
CKD-MBD: Chronic kidney disease–mineral and bone disorder
ESKD: End-stage kidney disease
FKC: Fresenius Kidney Care
iPTH: Intact parathyroid hormone
KDIGO: Kidney Disease: Improving Global Outcomes
KDOQI: Kidney Disease Outcomes Quality Initiative
Kru: Residual kidney function
MBD: Mineral and bone disorder
NEIRB: New England Independent Review Board
NKF: National Kidney Foundation
nPCR: Normalized protein catabolic ratio
PB: Phosphate binder
PD: Peritoneal dialysis
PDOPPS: Peritoneal Dialysis Outcomes and Practice Patterns Study
SD: Standard deviation
SO: Sucroferric oxyhydroxide
sP: Serum phosphorus

## References

[1] Karkar A, Wilkie M (2023). Peritoneal dialysis in the modern era. Perit Dial Int.

[2] US Renal Data System. 2022 USRDS annual data report: epidemiology of kidney disease in the United States. National Institutes of Health, National Institute of Diabetes and Digestive and Kidney Diseases, 2022. Accessed December 1, 2023. https://usrds-adr.niddk.nih.gov/2022.

[3] Covic A, Kothawala P, Bernal M, Robbins S, Chalian A, Goldsmith D (2009). Systematic review of the evidence underlying the association between mineral metabolism disturbances and risk of all-cause mortality, cardiovascular mortality and cardiovascular events in chronic kidney disease. Nephrol Dial Transpl.

[4] Lopes MB, Karaboyas A, Zhao J, Johnson DW, Kanjanabuch T, Wilkie M, Nitta K, Kawanishi H, Perl J, Pisoni RL, PDOPPS Steering Committee (2023). Association of single and serial measures of serum phosphorus with adverse outcomes in patients on peritoneal dialysis: results from the international PDOPPS. Nephrol Dial Transpl.

[5] Kidney Disease: Improving Global Outcomes (KDIGO) CKD-MBD Update Work Group. KDIGO 2017 clinical practice guideline update for the diagnosis, evaluation, prevention, and treatment of chronic kidney disease–mineral and bone disorder (CKD-MBD). Kidney Int Suppl. (2011). 2017;7(1):1–59. 10.1016/j.kisu.2017.04.001 PMC6340919 30675420

[6] Evenepoel P, Meijers BK, Bammens B, Viaene L, Claes K, Sprangers B, Naesens M, Hoekstra T, Schlieper G, Vanderschueren D, Kuypers D (2016). Phosphorus metabolism in peritoneal dialysis- and haemodialysis-treated patients. Nephrol Dial Transpl.

[7] Cupisti A, Gallieni M, Rizzo MA, Caria S, Meola M, Bolasco P (2013). Phosphate control in dialysis. Int J Nephrol Renovasc Dis.

[8] Rroji M, Seferi S, Cafka M, Petrela E, Likaj E, Barbullushi M, Thereska N, Spasovski G (2014). Is residual renal function and better phosphate control in peritoneal dialysis an answer for the lower prevalence of valve calcification compared to hemodialysis patients?. Int Urol Nephrol.

[9] Tonelli M, Pannu N, Manns B (2010). Oral phosphate binders in patients with kidney failure. N Engl J Med.

[10] Chiu YW, Teitelbaum I, Misra M, de Leon EM, Adzize T, Mehrotra R (2009). Pill burden, adherence, hyperphosphatemia, and quality of life in maintenance dialysis patients. Clin J Am Soc Nephrol.

[11] Floege J, Covic AC, Ketteler M, Rastogi A, Chong EM, Gaillard S, Lisk LJ, Sprague SM, PA21 Study Group (2014). A phase III study of the efficacy and safety of a novel iron-based phosphate binder in dialysis patients. Kidney Int.

[12] Kalantar-Zadeh K, Parameswaran V, Ficociello LH, Anderson L, Ofsthun NJ, Kwoh C, Mullon C, Kossmann RJ, Coyne DW (2018). Real-world scenario improvements in serum phosphorus levels and pill burden in peritoneal dialysis patients treated with sucroferric oxyhydroxide. Am J Nephrol.

[13] Perez L, You Z, Teitelbaum I, Andrews ES, Reddin R, Ramirez-Renteria L, Wilson G, Kendrick J (2022). A 6-month clinical practice pilot study of sucroferric oxyhydroxide on nutritional status in patients on peritoneal dialysis. BMC Nephrol.

[14] Clase CM, Norman GL, Beecroft ML, Churchill DN (2000). Albumin-corrected calcium and ionized calcium in stable haemodialysis patients. Nephrol Dial Transpl.

[15] Coyne DW, Ficociello LH, Parameswaran V, Anderson L, Vemula S, Ofsthun NJ, Mullon C, Maddux FW, Kossmann RJ, Sprague SM (2017). Real-world effectiveness of sucroferric oxyhydroxide in patients on chronic hemodialysis: a retrospective analysis of pharmacy data. Clin Nephrol.

[16] Kendrick JB, Zhou M, Ficociello LH, Parameswaran V, Mullon C, Anger MS, Coyne DW (2022). Serum phosphorus and pill burden among hemodialysis patients prescribed sucroferric oxyhydroxide: one-year follow-up on a contemporary cohort. Int J Nephrol Renovasc Dis.

[17] Berner T, Ferro C, Dieguez G, Metz S, Moore J, Szabo E, Kovesdy CP (2023). Real-world phosphate binder use among dialysis-dependent patients with CKD. Nephron.

[18] McCullough K, Port FK, de Sequera P, Rayner H, Pecoits-Filho R, Walpen S, Evenepoel P, Pisoni RL (2021). DOPPS Country investigators. European hemodialysis patient satisfaction with phosphate binders is associated with serum phosphorus levels: the Dialysis outcomes and practice patterns study. Clin Kidney J.

[19] Wang S, Alfieri T, Ramakrishnan K, Braunhofer P, Newsome BA (2014). Serum phosphorus levels and pill burden are inversely associated with adherence in patients on hemodialysis. Nephrol Dial Transpl.

[20] Fissell RB, Karaboyas A, Bieber BA, Sen A, Li Y, Lopes AA, Akiba T, Bommer J, Ethier J, Jadoul M, Pisoni RL, Robinson BM, Tentori F (2016). Phosphate binder pill burden, patient-reported non-adherence, and mineral bone disorder markers: findings from the DOPPS. Hemodial Int.

[21] Gong N, Xiao Z, Zhang F, Zhong X, He Y, Yi Z, Tang D, Yang C, Lin Y, Nie J, Ai J (2020). Duration of serum phosphorus control associated with overall mortality in patients undergoing peritoneal dialysis. Kidney Dis (Basel).

[22] Cernaro V, Calderone M, Gembillo G, Calabrese V, Casuscelli C, Lo Re C, Longhitano E, Santoro D (2023). Phosphate control in peritoneal dialysis patients: issues, solutions, and open questions. Nutrients.

[23] Li L, Liang W, Ye T, Chen Z, Zuo X, Du X, Qian K, Zhang C, Hu X, Li J, Wang L, Ma Z, Yao Y (2016). The association between nutritional markers and biochemical parameters and residual renal function in peritoneal dialysis patients. PLoS ONE.

[24] Shinaberger CS, Greenland S, Kopple JD, Van Wyck D, Mehrotra R, Kovesdy CP, Kalantar-Zadeh K (2008). Is controlling phosphorus by decreasing dietary protein intake beneficial or harmful in persons with chronic kidney disease?. Am J Clin Nutr.

