# Supplementary material for: Management of serum phosphorus over a 1-year follow-up in patients on peritoneal dialysis prescribed sucroferric oxyhydroxide as part of routine care: a retrospective analysis
Source: BMC Nephrol. 2024 Jun 17;25:197. doi: 10.1186/s12882-024-03633-8 (PMC11184799; doi:10.1186/s12882-024-03633-8)
Supplement: Supplementary file 1 — Supplementary material [file 12882_2024_3633_MOESM1_ESM.doc]

**Supplemental table 1** Comparison of mean quarterly sP levels among patients with a baseline sP >5.5 mg/dL that prescribed non-SO monotherapy vs. SO monotherapy

| **Population** | **Baseline** | **Follow-up** | | | | **Change from baseline to Q4** |
| --- | --- | --- | --- | --- | --- | --- |
|  | **–1Q** | **Q1** | **Q2** | **Q3** | **Q4** |  |
| Non-SO monotherapy (*n* = 2561) | 6.73 | 6.18 | 6.11 | 6.14 | 6.17 | 0.56 |
| SO monotherapy (*n* = 272) | 6.89 | 6.24 | 6.02 | 6.09 | 6.08 | 0.81 |

Values are presented as arithmetic means. sP, serum phosphorus; PB, phosphate binder; SO, sucroferric oxyhydroxide; Q, quarter.
